# Serum Organ-Specific Anti-Heart and Anti-Intercalated Disk Autoantibodies as New Autoimmune Markers of Cardiac Involvement in Systemic Sclerosis: Frequency, Clinical and Prognostic Correlates

**DOI:** 10.3390/diagnostics11112165

**Published:** 2021-11-22

**Authors:** Alida Linda Patrizia Caforio, Giacomo De Luca, Anna Baritussio, Mara Seguso, Nicoletta Gallo, Elisa Bison, Maria Grazia Cattini, Elena Pontara, Luna Gargani, Alessia Pepe, Corrado Campochiaro, Mario Plebani, Sabino Iliceto, Giovanni Peretto, Antonio Esposito, Lorenzo Tofani, Alberto Moggi-Pignone, Lorenzo Dagna, Renzo Marcolongo, Marco Matucci-Cerinic, Cosimo Bruni

**Affiliations:** 1 Cardiology Unit, Department of Cardiac, Thoracic, Vascular Sciences and Public Health, University of Padua, 35122 Padua, Italy; anna.baritussio@gmail.com (A.B.); elisa.bison@unipd.it (E.B.); mariagrazia.cattini@unipd.it (M.G.C.); elena.pontara@unipd.it (E.P.); sabino.iliceto@unipd.it (S.I.); 2 Unit of Immunology, Rheumatology, Allergy and Rare Diseases, IRCCS San Raffaele Hospital, Vita-Salute San Raffaele University, 20132 Milan, Italy; deluca.giacomo@hsr.it (G.D.L.); campochiaro.corrado@hsr.it (C.C.); dagna.lorenzo@hsr.it (L.D.); marco.matuccicerinic@unifi.it (M.M.-C.); 3 Department of Laboratory Medicine, University of Padua, 35122 Padua, Italy; mara.seguso@gmail.com (M.S.); nicoletta.gallo@aopd.veneto.it (N.G.); mario.plebani@unipd.it (M.P.); 4 Institute of Clinical Physiology, National Council of Research, 56124 Pisa, Italy; gargani@ifc.cnr.it; 5 Department of Medicine, Institute of Radiology, University of Padova, 35122 Padova, Italy; alessia.pepe@ftgm.it; 6 Unit of Arrhythmology, IRCCS San Raffaele Hospital, Vita-Salute San Raffaele University, 20132 Milan, Italy; peretto.giovanni@hsr.it; 7 Clinical and Experimental Radiology Unit, Expirimental Imaging Center, IRCCS San Raffaele Scientific Institute, Ospedale San Raffaele University, 20132 Milan, Italy; esposito.antonio@hsr.it; 8 Division of Rheumatology, Department of Experimental and Clinical Medicine, Department of Geriatric Medicine AOUC, University of Florence, 50121 Florence, Italy; lorenzo120787@gmail.com (L.T.); cosimo.bruni@unifi.it (C.B.); 9 Division of Internal Medicine Unit IV AOUC, Department of Experimental and Clinical Medicine, University of Florence, 50121 Florence, Italy; alberto.moggipignone@unifi.it; 10 Haematology and Clinical Immunology, Department of Medicine, University of Padua, 35122 Padua, Italy; renzo.marcolongo@tiscali.it

**Keywords:** myocarditis, autoimmunity, autoantibodies, systemic sclerosis, prognosis

## Abstract

Background: Heart involvement (HInv) in systemic sclerosis (SSc) may relate to myocarditis and is associated with poor prognosis. Serum anti-heart (AHA) and anti-intercalated disk autoantibodies (AIDA) are organ and disease-specific markers of isolated autoimmune myocarditis. We assessed frequencies, clinical correlates, and prognostic impacts of AHA and AIDA in SSc. Methods: The study included consecutive SSc patients (*n* = 116, aged 53 ± 13 years, 83.6% females, median disease duration 7 years) with clinically suspected heart involvement (symptoms, abnormal ECG, abnormal troponin I or natriuretic peptides, and abnormal echocardiography). All SSc patients underwent CMR. Serum AHA and AIDA were measured by indirect immunofluorescence in SSc and in control groups of non-inflammatory cardiac disease (NICD) (*n* = 160), ischemic heart failure (IHF) (*n* = 141), and normal blood donors (NBD) (*n* = 270). AHA and AIDA status in SSc was correlated with baseline clinical, diagnostic features, and outcome. Results: The frequency of AHA was higher in SSc (57/116, 49%, *p* < 0.00001) than in NICD (2/160, 1%), IHF (2/141, 1%), or NBD (7/270, 2.5%). The frequency of AIDA was higher (65/116, 56%, *p* < 0.00001) in SSc than in NICD (6/160, 3.75%), IHF (3/141, 2%), or NBD (1/270, 0.37%). AHAs were associated with interstitial lung disease (*p* = 0.04), history of chest pain (*p* = 0.026), abnormal troponin (*p* = 0.006), AIDA (*p* = 0.000), and current immunosuppression (*p* = 0.01). AHAs were associated with death (*p* = 0.02) and overall cardiac events during follow-up (*p* = 0.017). Conclusions: The high frequencies of AHA and AIDA suggest a high burden of underdiagnosed autoimmune HInv in SSc. In keeping with the negative prognostic impact of HInv in SSc, AHAs were associated with dismal prognosis.

## 1. Introduction

Heart involvement (HInv) is common in systemic sclerosis (SSc) and may be primary or secondary to kidney and/or pulmonary vascular/interstitial disease [1]. It is often clinically silent, and when symptomatic, carries a poor prognosis, accounting for one third of total deaths [2]. Primary HInv is associated with a variable phenotype: clinical presentations include dyspnoea, palpitations, arrhythmias, chest pain, heart failure (HF) with depressed left ventricular ejection fraction (LVEF), and diastolic dysfunction, although most patients are asymptomatic at early stages [3,4]. SSc may have a high arrhythmic burden [5], with a 5% sudden death rate in patients with both skeletal and cardiac muscle diseases [6].

The pathophysiology of primary HInv is poorly understood and may relate to dysfunctional and/or structural micro vessel-vasculopathy, repeat focal ischaemic injury, irreversible fibrosis, and/or myocarditis [7,8]; the degree of both myocardial inflammation and fibrosis has been associated with poor outcomes [9]. Biopsy-proven myocarditis is increasingly reported in SSc [10,11] in association with negative prognosis [11].

Clinical and non-invasive red flags for myocarditis in SSc are similar to those observed in organ-specific autoimmune myocarditis [12], as well as in myocarditis observed in the context of other systemic immune-mediated diseases (SIDs) [13,14]. These features include unexplained increases in troponin(s) [15], left ventricular systolic dysfunction and/or left ventricular diastolic dysfunction [16], and non-ischaemic abnormal cardiac magnetic resonance (CMR) tissue patterns in both symptomatic and asymptomatic cases [17,18,19]. Early recognition of myocarditis in SSc is clinically relevant, since myocarditis is an indication for upgrading immunosuppressive treatment [10,11,12,13,20].

Serum anti-heart (AHA) and anti-intercalated disk antibodies (AIDA) are organ and disease-specific early markers of isolated (i.e., organ-specific) autoimmune myocarditis within the entire spectrum of its clinical presentations. They identify patients and symptom-free relatives at risk of disease progression but are very uncommon in other non-immune-mediated cardiac diseases, including coronary artery disease, or in normal subjects [12,21,22,23,24]. In addition, we have recently reported a high frequency and high specificity of AHA and AIDA for cardiac involvement in sarcoidosis [25].

It is currently unknown whether or not AHA and AIDA are found in SSc and whether they are associated with HInv. In this study, we assessed frequency, clinical, diagnostic, and prognostic correlates of serum AHA and AIDA in SSc.

## 2. Methods

### 2.1. Study Patients and Data Collection

The study included consecutive SSc patients with clinically suspected HInv, followed up at the Rheumatology Department, Azienda Ospedaliera Universitaria Careggi (Florence, Italy), and at the Unit of Immunology, Rheumatology, Allergy, and Rare Diseases, IRCCS San Raffaele Hospital (Milan, Italy). Clinically suspected HInv was defined as possessing one or more of the following features: palpitation, dyspnoea or syncope, abnormal troponin I or natriuretic peptides levels, abnormal 12-lead ECG, 24-h ECG Holter monitoring, or echocardiographic findings [4]. All patients underwent CMR. Clinical and diagnostic data at diagnosis and follow-up were retrospectively reviewed. Data collection included the following:

1. Patient history and symptoms: disease duration from first non-Raynaud’s phenomenon, smoking exposure (past or current), New York Heart Association (NYHA) functional class for dyspnoea, history of chest pain, palpitations, and syncope;
2. Full rheumatological assessments: fulfillment of the EULAR/ACR 2013 classification criteria [26], specifying when this was obtained through a very early diagnosis of systemic sclerosis (VEDOSS) features only [27]; modified Rodnan skin score (mRSS) and type of skin involvement (limited or diffuse) according to LeRoy classification [28]; nailfold video capillaroscopy (NVC) pattern [29]; presence of interstitial lung disease (ILD) on high resolution computed tomography (HRCT) and its functional assessment with pulmonary function tests, including forced vital capacity (FVC) and diffusion lung capacity of carbon oxide (DLco); history of digital ulcers (DUs) or current DUs; gastro-intestinal involvement; history of renal crisis; ongoing vasodilating; and/or ongoing and past immunosuppressive medications;
3. Cardiological workup: evaluation of traditional cardiovascular risk factors; standard 12-lead ECG; 24 h ECG Holter monitoring; standard transthoracic echocardiography; morpho functional and tissue characterization by CMR. The following CMR parameters were included: left and right chamber volumes; left and right ventricular function; presence and pattern of late gadolinium enhancement (LGE); qualitatively evaluated T2 short T inversion recovery (STIR) images for increased signal at myocardial and pericardial level; and presence of pericardial effusion;
4. Laboratory parameters: anti-nuclear antibodies (ANA); anti-topoisomerase I (ATA) antibodies; anti-centromere antibodies (ACA); anti-RNA polymerase III antibodies and other rare SSc-related antibodies by standard methods according to local laboratory; type B pro-natriuretic peptide (NT-proBNP); high-sensitive troponin I or T; and creatinine clearance.

The study protocol followed the ethical guidelines of the Declaration of Helsinki and obtained Institutional Review Board approval at the referral Hospitals of the following: Ethics Committee of Padova, protocol number 0027841, date 6 May 2020; Ethics Committee of San Raffaele Hospital, protocol Immunoradar Dsan number 1178/9, date 8 March 2018; Ethics Committee Toscana Area Vasta Nord-Ovest, protocol number 2849, date 5 October 2015.

All participants provided written informed consent. ALPC has full access to all the data in the study and takes responsibility for its integrity and data analysis.

### 2.2. Serum AHA and AIDA Testing by Indirect Immunofluorescence (IF)

AHA and AIDA were detected by indirect immunofluorescence (IF) at 1/10 dilution on 4 µm thick unfixed fresh frozen cryostat sections of blood group O normal human atrium and skeletal muscle [21,22,23,24,25,30]. Two sera were used as standard positive and negative controls and titrated in every assay. All sera were read blindly from clinical diagnosis against these standards by using a fluorescence microscope (Zeiss Axioplan 2 imaging, Zeiss, New York, NY, USA). An additional positive control serum was titrated in order to assess reproducibility. End point titres for this serum were reproducible within one double dilution in all assays [21,22,23,24,25,30]. The frequency of AHA and of AIDA in SSc was compared with that observed in previously established control groups of non-inflammatory cardiac disease (NICD) (*n* = 160, 80 male, aged 37 ± 17 years, of whom *n* = 55 with other rheumatic-related heart diseases without myocarditis, *n* = 67 hypertrophic cardiomyopathy, and *n*= 38 congenital defects), ischemic heart disease (*n* = 141, 131 male, aged 44 ± 14 years), and normal individuals (*n* = 270, 123 male, age 35 ± 11) [21,22,23,24,29]. These control sera were obtained with informed consent from patients admitted to the hospital and were tested blindly from diagnosis at the time of description and validation of the IF assay [21,22,23,24,25,30].

### 2.3. Prognostic Evaluation

Patients were regularly followed at the caring Rheumatology centre. Prognostic data were retrospectively collected from patients’ charts. If patients were lost to follow-up, a telephone contact with the patient/general practitioner was made to collect or confirm the acquired data. The following events were considered as outcomes: (1) death and (2) cardiac worsening, defined as the development of at least one condition of the following: pulmonary hypertension (PH) according to the 2015 ESC/ERS guidelines [31]; right HF; left HF; non-sustained ventricular tachycardia (nSVT) on 24 ECG Holter monitoring, positioning of an implantable cardioverter defibrillator (ICD); development of angiographically proven coronary artery disease (CAD). Data were collected until death or last available follow-up date. A mean follow-up for each patient was then calculated.

### 2.4. Statistical Analysis

The results for quantitative measures are given as mean ± SD or as median (interquartile range) for variables deviating from normal distribution, and qualitative measures are given as frequency (percentage). Quantitative variables were compared by one-way analysis of variance, Student’s *t*-test if normally distributed, or by Mann–Whitney test if deviating from normal distribution. Qualitative measures were compared by χ^2^ test or Fisher’s exact test as appropriate. Differences in actuarial survival curves according to the studied variables were assessed by Mantel–Haenszel log-rank test and graphically presented by Kaplan–Meier curve analysis. Logistic regression tested the association between predictors and binary outcomes (expressed via Odds Ratio—OR), while Cox-regression was used to calculate the Hazard Ratio (HR) of the predictor for events over time; both measures were calculated and presented with their 95% Confidence Interval (95% CI). Adjusted *p*-values less than 0.05 were considered to indicate statistical significance. All statistical analyses were performed by using the SPSS software version 25.0 (SPSS, Inc, Chicago, IL, USA, 2017).

## 3. Results

### 3.1. Baseline Features of the Study Population

A total of 116 SSc patients were recruited. The clinical and diagnostic features at baseline are presented in Table 1.

Briefly, the majority of patients were females (83.6%), with mean age of 53 ± 13 years and a median disease duration of 7 years, and half of them had past or current smoking exposure. Clinically, two-thirds of the population presented a limited cutaneous SSc form, and about one-third had DUs at any time of the disease course, while ILD on HRCT was present in 52% of patients. Cardiac symptoms, including syncope, palpitation, chest pain, and advanced NYHA class (≥class 3), were reported in a minority of patients. On 24 h Holter ECG, supraventricular and ventricular ectopic beats were a common finding, but while most patients had a low daily number of ectopies, none had complex or repetitive ventricular tachyarrhythmia. Although biventricular systolic function, as assessed by standard echocardiography and CMR, was within the normal range, tissue characterization by CMR showed non-ischemic LGE patterns in 40.5% of patients and myocardial oedema on T2 STIR sequences in 13%. Pericardial effusion was an uncommon finding (19%). Overall, median NT-proBNP and high sensitivity troponin I or T were within the normal range, with some (11% and 4% respectively) patients having abnormal values according to the local laboratory. The vast majority of patients was ANA positive, with ATA and ACA positivity representing the SSc specific-antibody subset in two-thirds of the population. One-third was on immunosuppressive therapy at the time of study inclusion, mycophenolate mofetil being the most commonly used drug (44%), with a sizable proportion (22%) of patients on biological agents; a minority of patients (13%) had been previously treated with steroids.

### 3.2. AHA and AIDA: Frequency and Associations with Baseline Clinical and Diagnostic Features

**Figure 1:**
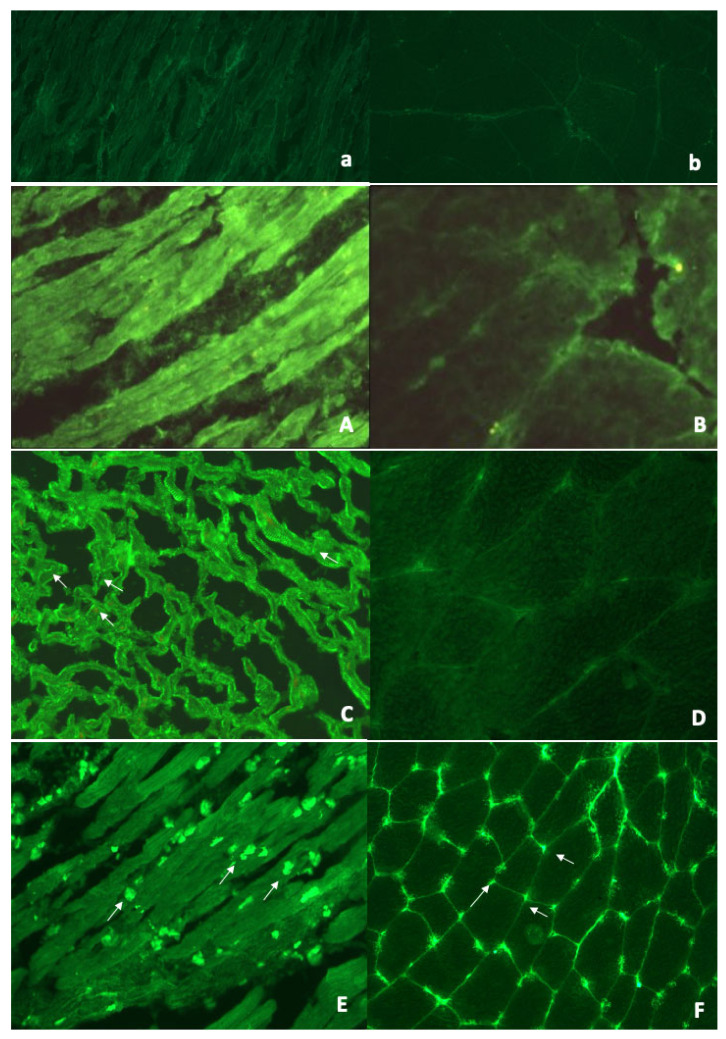
Representative Anti-Heart Auto-antibodies (AHA) and Anti-Intercalated Disk Autoantibodies (AIDA) patterns by indirect immunofluorescence test. Negative AHA, AIDA, and ANA control serum pattern on human heart tissue (panel (**a**), ×200) and on human skeletal muscle (panel (**b**), ×400). *Organ-specific AHA pattern*: panel (**A**) on human heart tissue: strong diffuse cytoplasmic staining of cardiac myocytes (organ-specific AHA pattern) (×400); panel (**B**) (×400) on human skeletal muscle tissue: negative. *Organ-specific AHA and AIDA pattern*: panel (**C**) strong linear staining of the intercalated disks (AIDA pattern) (white arrows) and associated organ-specific AHA diffuse and fine striational pattern (×400); panel (**D**) (×400) on human skeletal muscle tissue: negative. *Organ-specific AHA and ANA pattern*: panel (**E**) on human heart tissue: strong diffuse cytoplasmic staining of cardiac myocytes (organ-specific AHA pattern) and associated antinuclear antibody (ANA) (white arrows) (×200); panel (**F**) (×200) on human skeletal muscle tissue: negative for AHA and positive for ANA (white arrows). Note the intracellular location of ANA on human heart and the peripheral location of ANA on skeletal muscle.

Sera from all 116 patients, taken at the time of study evaluation, were studied blindly from clinical diagnosis. Organ-specific and cross-reactive AHA patterns were classified as described [21,22,23,24,29]. Representative examples of organ-specific AHA and AIDA patterns are shown in Figure 1.

The AHA patterns have been previously described [21,22,23,24,25,30]. As shown in Figure 1, organ-specific AHA provided diffuse cytoplasmic staining with or without additional fine striational staining of atrial myocytes but were negative on skeletal muscle. Cross-reactive 1 or partially organ-specific AHA (not shown) provided fine striational staining on atrium and were negative or only weakly stained skeletal muscle; cross-reactive 2 AHA (not shown) provided a broad striational pattern on longitudinal sections of heart and skeletal muscle [21,22,23,24,25,30]. The cardiac-specificity and cross-reactivity with skeletal muscle of the AHA patterns were confirmed by absorption studies with relevant tissues [21]. AIDA provided linear staining of the intercalated disks between cardiac myocytes [30].

The frequency of organ-specific AHA was higher in SSc (57/116, 49%) than in NICD (2/160, 1%), IHF (2/141, 1%), or NBD (7/270, 2.5%) (*p* < 0.00001 for all comparisons). Organ-specific AHA scores in SSc patients were weak in 28 (24.1%), positive in 20 (17.2%), and strong positive in 9 (7.8%); no SSc sera tested positive for cross-reactive AHA types. The frequency of AIDA was higher (65, 56%, *p* < 0.00001) in SSc than in NICD (6/160, 3.75%), IHF (3/141, 2%), or NBD (1/270, 0.37%) (*p* < 0.00001 for all comparisons). AIDA scores in SSc were weak in 30 (25.9%), positive in 31 (26.7%), and strong positive in 4 (3.4%).

Associations of AHA and AIDA status with clinical and diagnostic features are shown in Table 2 and Table 3, respectively.

AHA positivity was more prevalent in patients with past and/or current smoking exposure (*p* = 0.009) and tended to be more common in female SSc patients (*p* = 0.09). There was a higher frequency of AHA positivity in SSc patients with ILD detected on HRCT (*p* = 0.041), with current immunosuppressive therapy (*p* = 0.011), in those with current prostanoid therapy (*p* = 0.006) and a trend for an association with ATA positivity (*p* = 0.057) and female gender (*p* = 0.09). From a cardiologic perspective, AHAs were associated with a history of chest pain (*p* = 0.026), higher high-sensitivity troponin I levels (*p* = 0.006), AIDA (*p* < 0.001), and tended to be associated and pericardial oedema on CMR (*p* = 0.053).

Similarly, AIDA positive status was more frequent in patients with past and/or current smoke exposure (*p* = 0.007) and tended to be more common in female SSc patients (*p* = 0.067). From a rheumatological point of view, AIDA positive patients presented more frequently with NVC SSc pattern (*p* = 0.01) with a trend for a more advanced NVC pattern (*p* = 0.056) and for DUs at any time since SSc diagnosis (*p* = 0.071), as well as with a higher frequency of current prostanoid therapy (*p* = 0.002). A lower number of AIDA positive patients had ventricular ectopic beats on 24 h Holter monitoring (*p* = 0.007).

Conversely, both AHA and AIDA were not differently distributed according to the presence of systemic arterial hypertension, diabetes mellitus, dyslipidaemia, PH, Raynaud’s phenomenon, betablocker, calcium antagonist or antiarrhythmic drugs, sildenafil, bosentan, statins, and previous immunosuppressive or steroid therapy (not shown). The remaining baseline clinical and diagnostic features (echocardiographic, HRCT, and CMR), including LVEF and RVEF, were similar in AHA and in AIDA positive and negative patients (not shown).

Given the higher prevalence of both AIDA and AHA in patients with smoking exposure, we considered the possible association between tobacco and antibody positivity. After adjustment for age and gender, smoking exposure was a statistically significant predictor of AHA positivity (OR 2.83, 95% CI 1.31–6.13, *p* = 0.008) and AIDA positivity (OR 2.87, 95% CI 1.32–6.26, *p* = 0.008). These results were not confirmed when targeting positive/strong positive antibody scores, both for AHA (OR 1.85, 95% CI 0.79–4.41, *p* = 0.158) and AIDA (OR 1.71, 95% CI 0.766–3.84, *p* = 0.189), hypothesizing a relationship between the toxic agent and a weak level antibody production in a population with autoimmune disease.

When the subgroups of patients with positive/strong positive scores of AHA (*n* = 29) or AIDA (*n* = 35) were compared to the respective rest of the study population, no meaningful or significant changes in the distributions of prevalence shown in Table 2 and Table 3 were detected (data not shown).

### 3.3. Follow-Up and Prognostic Evaluation

Follow-up data were available for 115 of the 116 patients is shown in Table 4, one patient was lost to follow-up. After a mean follow-up of 43 ± 28 months, five deaths were recorded, and 15 patients developed a cardiac event, particularly one right HF, two left HF, one PH, three nSVT, three ICD implants, eight other arrhythmias, and three CAD. Five patients developed more than one event during follow-up.

**Figure 2:**
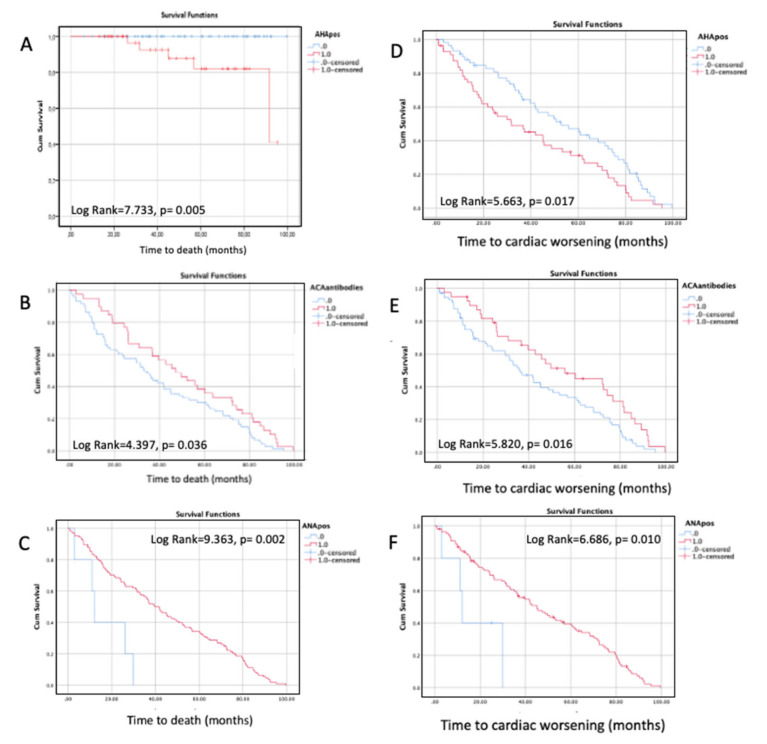
Survival curves according to auto-antibodies status. (**A**) Kaplan–Meier survival curves in systemic sclerosis (SSc) patients according to Anti-Heart Autoantibodies (AHA) status. AHA positive SSc patients have lower survival (*p* = 0.005). (**B**) Kaplan–Meier curves comparing survival in systemic sclerosis (SSc) patients as a function of Anti-Centromere Autoantibody (ACA); ACA positive SSc patients have better survival (*p* = 0.036). (**C**) Anti-Nuclear Autoantibody (ANA) status. ANA positive SSc patients have better survival (*p* = 0.002). Kaplan–Meier curves in systemic sclerosis (SSc) comparing time to cardiac worsening as a function of (**D**) Anti-Heart Autoantibodies (AHA), AHA positive SSc patients have lower survival free from cardiac worsening (*p* = 0.017); (**E**) Anti-Centromere Autoantibody (ACA), ACA positive SSc patients have higher survival free from cardiac worsening (*p* = 0.016); (**F**) Anti-Nuclear Autoantibody (ANA) status. ANA positive SSc patients have higher survival free from cardiac worsening (*p* = 0.010). In all curves, 0 = negative antibody status, 1 = positive antibody status.

All outcome measures were similarly distributed in AHA positive and AHA negative patients. However, the frequency of death was higher among AHA positive patients (8.9% vs. 0%, *p* = 0.021); in particular, all five deaths occurred in AHA positive patients. AHA positive status was also associated with worse survival by log-rank test (Figure 2A) (*p* = 0.005). While the overall AHA positivity was not a significant predictor of death on univariate Cox regression (*p* = 0.222), its positive/strong positive scores (*n* = 29 patients) resulted in a significant predictor of deaths over time (HR 12.69, 95% CI 1.41–113.89, *p* = 0.023).

Conversely to the AHA positive status, ACA positive and ANA positive statuses were associated with better survival by log-rank test (*p* = 0.036; *p* = 0.002) (Figure 2B top and bottom, respectively). AHA positive status was also associated with lower survival free from cardiac worsening by log-rank test (*p* = 0.017) (Figure 2C, top). Conversely to the AHA positive status, ACA positive and ANA positive statuses were associated with higher survival free from cardiac worsening by log-rank test (*p* = 0.016; *p* = 0.01) (Figure 2C, middle and bottom).

AIDA positivity was not associated with any prognostic outcome (Supplementary Table S1).

## 4. Discussion

### 4.1. Frequency and Significance of AHA and AIDA in SSc

A novel finding of this cross-sectional study is the detection of organ-specific AHA and AIDA at a higher frequency in SSc than in a large control cohort of other non-immune-mediated cardiac diseases, including coronary artery disease, and in normal subjects [12,21,22,23,24,25]. These markers are found in 60–80% of patients with biopsy-proven organ-specific autoimmune myocarditis/inflammatory cardiomyopathy in its entire spectrum of clinical presentations (fulminant, acute, subacute, chronic heart failure, pseudo infarct, and arrhythmic presentation) [21,22,23,24] and in clinically suspected myocarditis temporally associated with COVID-19 [32]. In addition, we have recently reported a high frequency and high specificity of AHA and AIDA for cardiac involvement in sarcoidosis [25]. Thus, AHA and AIDA provide organ and disease-specific markers of immune-mediated myocarditis, either isolated (organ-specific) or in the context of SIDs, such as sarcoidosis. In keeping with this interpretation, the frequencies of AHA (49%) and AIDA (56%) reported here were similarly high in an SSc cohort with established disease and a median disease duration of 7 years, indicating a potentially high burden of underdiagnosed clinically pauci-symptomatic autoimmune cardiac involvement. In fact, cardiac symptoms, including syncope, palpitation, chest pain, and advanced NYHA class, were reported in a minority of patients. Left and right ventricular pump functions as assessed, at the time of serum antibody testing, by standard echocardiography and CMR were within the normal range. However, tissue characterisation by CMR showed a non-ischemic LGE pattern in 40.5% of patients and myocardial oedema on T2 sequences in 13% of patients; the CMR findings were compatible with the diagnosis of clinically suspected myocarditis and rule out previous myocardial infarction. Since CMR accuracy is low in chronic heart failure and arrhythmia scenarios [12] and endomyocardial biopsy data are not available in this SSc cohort, it is possible that the frequency of myocarditis is higher than 40%, as suggested by the presence of AHA and/or AIDA in up to 56% of patients. Although AHA and AIDA are associated with SSc, future studies are needed in order to clarify whether or not they have a direct pathogenic role in SSc associated immune-mediated myocardial damage, as suggested for these specificities in organ-specific myocarditis [33]. In previous studies on AHA, it was demonstrated that Western blot [34] and ELISA [12] are more sensitive than IF in recognizing autoantibodies directed against specific heart autoantigens, which for AHA include alpha and beta myosin heavy chain isoforms [34]. On the other hand, IF, the standard autoimmune serology technique, is best suited for the detection of multiple autoantibody reactions simultaneously [21] on a sizable number of patients and controls. The present IF findings show polyclonal humoral autoimmune reactivity against myocardial targets in SSc sera, including AHA (which are directed against alpha and beta myosin heavy chains and other yet unidentified autoantigens [34]) and AIDA (directed against yet unknown autoantigens). Further studies should focus on the identification of the autoantigen(s) responsible for these IF patterns in SSc.

### 4.2. AHA and AIDA: Significance of Associations with Baseline Clinical and Diagnostic Features

AHA positive status was associated with smoking exposure and ILD. This may suggest an adjuvant effect of environmental toxic agents associated with tabagism on the autoimmune response to the myocardium. Previous studies showed opposite results when comparing smoking exposure and ATA positivity, possibly also reflecting the different structure and location of the target antigen [35].

AHA positivity was more prevalent in patients with a history of chest pain and higher troponin levels at baseline. These associations suggest underlying myocarditis rather than epicardial coronary artery disease. In fact, AHA positivity at baseline was not associated with coronary artery disease risk factors, i.e., systemic arterial hypertension, diabetes mellitus, and dyslipidaemia; with typical chest pain at serum evaluation; and with cardioactive drug use, i.e., betablocker, calcium antagonists, antiarrhythmics, and statins. In addition, CMR excluded an ischemic LGE pattern in all patients. The history of chest pain and the abnormal troponins are also unlikely to reflect PH, since AHA positivity was not associated with PH or with concomitant use of PDE5-inhibitors or anti-endothelin drugs. Conversely, AHA positivity was associated with current IS therapy and tended to be associated with female gender, current prostanoid therapy, and ATA positivity, suggesting cardiac involvement in severe, active disease in females.

AIDA positive status was also associated with previous or active smoker status and tended to be more prevalent in the female gender and ATA positivity, similarly to AHA positivity. Again, as for AHA, the AIDA status was not associated with the findings of systemic arterial hypertension, diabetes mellitus, dyslipidaemia, PH, Raynaud’s phenomenon, betablocker, calcium antagonist or antiarrhythmic drugs, sildenafil, bosentan, and statins. The relation of AIDA with current prostanoid therapy and with SSc patterns on NVC and the trend towards an association with digital ulcers at any time since diagnosis may suggest cardiac involvement in a subset of SSc with more pronounced peripheral vascular involvement. A lower number of AIDA positive patients had ventricular ectopic beats on 24 h Holter monitoring. In autoimmune organ-specific myocarditis, AIDAs are associated with high arrhythmia burden, but IS is highly effective at reducing arrhythmias [24]; therefore, the inverse association between arrhythmia and AIDA in SSc may be explained by the concomitant use of IS.

### 4.3. AHA, AIDA, and Prognostic Outcomes

Among the studied autoantibody markers, only AHA positivity was associated with death during follow-up and resulted as a significant predictor of mortality over time, when positive/strong positive results were considered as meaningful. In addition, only AHA positivity was associated with lower survival free from cardiac worsening. Conversely to AHA positivity, ACA positive and ANA positive status was associated with better survival and with higher survival free from cardiac worsening. These findings suggest that AHA positivity could be a novel non-invasive marker of negative prognosis, associated with primary HInv in SSc. This is also in keeping with the recognized independent prognostic role of primary HInv in SSc [13].

### 4.4. Study Limitations

The cross-sectional study design does not allow clarifying the time evolution of primary HInv in the context of SSc natural history. Similarly, prospective longitudinal studies are needed to define the diagnostic accuracy of AHA as an early predictor of primary HInv and its related morbidity and mortality as well as its incremental value over conventional cardiac biomarkers. The lack of endomyocardial biopsy does not allow defining the correlation of AHA positivity with concomitant histologically proven infectious-negative autoimmune myocarditis. Finally, the retrospective nature of data collection, together with the low number of cardiac events, represents a study limitation that did not allow us to test the predictive value of AHA antibodies adjusted for other confounders.

## 5. Conclusions

The high frequencies of AHA and AIDA reported here suggest a potentially high burden of underdiagnosed clinically pauci-symptomatic autoimmune HInv in established SSc. AHA positivity seems to be a novel autoimmune marker of HInv in severe, diffuse, and active SSc in females associated with negative prognosis.

## Figures and Tables

**Table 1:** Demographic, clinical, imaging, and immunological characteristics of SSc patients.

	*n* = 116
Female gender, *n* (%)	97 (83.6)
Age, years	53 ± 13
Median disease duration (range), years	7 (0–41)
Previous smoker, *n* (%)	55 (47)
SSc types (limited; diffuse; sine scleroderma) *n* (%)	77 (66); 36 (31); 3 (3)
mRSS (data available, *n* = 105), median, (range)	2 (0–35)
VEDOSS status (yes) *n* (%)	28 (24)
Digital ulcers (at any time, previous or current) (*n* = 115)	42 (36)
NVC pattern (*n* = 113): Early, *n* (%); Active, *n* (%); Late, *n* (%)	31 (27); 48 (42); 23 (21)
ILD on HRCT (yes), (*n* = 105), *n* (%)	55 (52)
Syncope (yes), *n* (%)	2 (2)
Palpitation (yes), *n* (%)	19 (16)
Chest pain (yes), *n* (%)	5 (4)
Advanced NYHA class (III and IV) at diagnosis, *n* (%)	8 (6)
Patients with supraventricular ectopic beats on ECG Holter monitoring (*n* = 78), *n* (%)	53 (67.9)
Patients with supraventricular tachycardia on ECG Holter monitoring (*n* = 77), *n* (%)	12 (15.5)
Patients with ventricular ectopic beats on ECG Holter monitoring (*n* = 77), *n* (%)	51 (66)
Patients with ventricular couplets on 24 h ECG Holter monitoring (*n* = 78), *n* (%)	15 (19%)
Ventricular ectopic beats total number on 24-h ECG Holter monitoring (*n* = 67), median (range)	4 (0–32,024)
LVEF Echo, (*n* = 113), mean ± SD	63 ± 6
TAPSE, mm (*n* = 84), mean ± SD	22 ± 3
PAPs on Echo, mmHg, (*n* = 68), mean ± SD	26 ± 6
LVEF% at CMR, (*n* = 116), mean ± SD	65 ± 7
RVEF% at CMR, (*n* = 112), mean ± SD	65 ± 7
Presence of LGE by CMR, (*n* = 114), *n* (%)	47 (40.5)
Presence of myocardial oedema by T2 STIR, (*n* = 115), *n* (%)	15 (13)
Oedema distribution on CMR, (*n* = 13) concordant with LGE site, another site from LGE, *n* (%)	7 (54); 6 (46)
Presence of pericardial oedema by T2 STIR, (*n* = 115), *n* (%)	4 (3.5)
Pericardial effusion by CMR, (*n* = 115), *n* (%)	22 (19)
NT-proBNP, (*n* = 87), median (range)	110 (10–2374)
Creatinine clearance, (*n* = 76), mean ± SD	94 ± 29
High sensitivity troponin I, (*n* = 55), median (range)	0.26 (0–490)
AHA positivity, *n* (%)	57 (49)
AIDA positivity, *n* (%)	65 (56)
ATA positivity, (*n* = 114) *n* (%)	41 (36)
ACA positivity, (*n* = 113) *n* (%)	39 (34.5)
ANA positivity, (*n* = 114) *n* (%)	109 (95.6)
Currently on IS (yes), *n* (%)	36 (31)
Current IS type, (*n* = 36), *n* (%): methotrexate; azathioprine; cyclophosphamide; mycophenolate mofetil; biologic agents	8 (22);2 (5.5); 2 (5.5);16 (44%); 8 (22)
Past IS (yes), *n* (%)	13 (11)
Past IS type, (*n* = 13), *n* (%): methotrexate; azathioprine; cyclophosphamide; mycophenolate mofetil; biologic agents	4 (31); 1 (7.6); 5 (38); 1 (7.6%); 2 (15)
Past steroid therapy, *n* (%):	15 (12.9)

Data are expressed as *n* (%) and mean ± SD or median (IQR). ACA, anticentromere antibody; AHA, anti-heart antibody; AIDA, anti-intercalated disk antibody; ANA, anti-nuclear antibody; CMR, cardiovascular magnetic resonance; ECG, electrocardiogram; Echo, echocardiography; HRCT, high resolution computed tomography; ILD, interstitial lung disease; IS, immunosuppression; LGE, late gadolinium enhancement; LVEF, left ventricular ejection fraction; mRSS, modified Rodnan skin score; NVC, nailfold videocapillaroscopy; NYHA, New York Heart Association; PAPs, pulmonary artery pressure in systole; RVEF, right ventricular ejection fraction; ATA, topoisomerase I; SSc, systemic sclerosis; STIR, short T inversion recovery; TAPSE, tricuspid Annulus Plane Systolic Excursion; VEDOSS, very early diagnosis of systemic sclerosis.

**Table 2:** Demographic, clinical, imaging, and immunological characteristics of patients according to AHA positivity.

	AHA Positive *n* = 57	AHA Negative *n* = 59	*p*-Value
Age, mean ± SD, years	51.8 ± 13	54 ± 13	0.814
Female gender, *n* (%)	51 (89.5)	46 (78)	0.097
Smoking exposure, *n* (%)	34 (59.6)	21(35.6)	0.009
SSc types (limited; diffuse; sine scleroderma) *n*	37/18/2	40/18/1	0.93
VEDOSS status (yes) *n* (%)	11 (19.3)	17 (28.8)	0.230
Digital ulcers (at any time, previous or current) (*n* = 115)	25 (43.9)	17 (29.3)	0.102
NVC pattern (*n* = 113): Early; Active, Late;	13/14/26	18/9/22	0.2208
ILD on HRCT (yes), (*n* = 105), *n* (%)	33 (62.3)	22 (42.3)	0.041
Syncope (yes), *n* (%)	1 (1.8)	1 (1.7)	>0.999
Palpitation (yes), *n* (%)	10 (17.5)	9 (15.3)	0.733
Chest pain (yes), *n* (%)	5 (8.8)	0 (0)	0.026
NYHA class (I-II, III, and IV) at diagnosis (*n* = 115)	51/5/1	56/2/0	0.285
Presence of LGE by CMR, (*n* = 114), *n* (%)	25 (44.6)	22 (37.9)	0.597
Presence of myocardial oedema by T2 STIR, (*n* = 115), *n* (%)	9 (16.4)	6 (10.2)	0.320
Presence of pericardial oedema by T2 STIR, (*n* = 115), *n* (%)	4 (7.1)	0 (0)	0.053
Pericardial effusion by CMR, (*n* = 115), *n* (%)	13 (23.2)	9 (15.3)	0.345
Patients with ventricular ectopic beats on ECG Holter monitoring (*n* = 77), *n* (%)	23 (63.9)	28 (68.3)	0.680
High sensitivity troponin I, (*n* = 55), median (range)	63 (10–121)	25 (1–59)	0.006
ATA positivity, (*n* = 114) *n* (%)	25 (44.6)	16 (27.6)	0.057
AIDA positivity, *n* (%)	48 (84)	17 (28.8)	<0.001
ACA positivity, (*n* = 113) *n* (%)	15 (27.3)	24 (41.4)	0.118
ANA positivity, (*n* = 114) *n* (%)	52 (92.9)	57 (98.3)	0.204
Currently on IS (yes), *n* (%)	24 (42.1)	12 (20.3)	0.011
Currently on Prostanoids (*n* = 115) (yes), *n* (%)	19 (33.9)	11 (18.6)	0.062

Data are expressed as *n* (%) and mean ± SD (or median, IQR). See Table 1 for abbreviations.

**Table 3:** Demographic, clinical, imaging, and immunological characteristics of patients according to AIDA positivity.

	AIDA Positive *n* = 65	AIDA Negative *n* = 51	*p*-Value
Age, years	52.6 ± 12	53.2 ± 14	0.812
Female gender, *n* (%)	58 (89.2)	39 (76.5)	0.067
Smoking exposure, *n* (%)	38 (58.5)	17(33.3)	0.004
SSc types (limited; diffuse; sine scleroderma) *n*	45/19/1	32/17/2	0.619
VEDOSS status (yes) *n* (%)	14 (21.5)	14 (27.5)	0.460
Digital ulcers (at any time, previous or current) (*n* = 115)	28 (43.8)	14 (27.5)	0.071
NVC pattern (*n* = 113): Early; Active, Late.	17/15/29	14/8/19	0.056
ILD on HRCT (yes), (*n* = 105), *n* (%)	34 (57.6)	21 (45.7)	0.226
Syncope (yes), *n* (%)	1 (1.5)	1 (2)	>0.999
Palpitation (yes), *n* (%)	7 (10.8)	12 (23.5)	0.065
Chest pain (yes), *n* (%)	4 (6.2)	1 (2)	0.388
NYHA class (I-II, III, and IV) at diagnosis (*n* = 115)	58/6/0	49/1/1	0.142
Presence of LGE on CMR, (*n* = 114), *n* (%)	25 (53.2)	22 (46,8)	0.566
Presence of myocardial oedema by CMR, (*n* = 115), *n* (%)	10 (15.6)	5 (10.0)	0.371
Presence of pericardial oedema by CMR, (*n* = 115), *n* (%)	4 (6.2)	0 (0)	0.135
Pericardial effusion by CMR, (*n* = 115), *n* (%)	14 (21.5)	8 (16.0)	0.485
Patients with ventricular ectopic beats on ECG Holter monitoring (*n* = 77), *n* (%)	22 (52.4)	29 (82.3)	0.007
ATA positivity, (*n* = 114) *n* (%)	27 (42.9)	14 (27.5)	0.080
ACA positivity, (*n* = 113) *n* (%)	19 (30.6)	20 (39.2)	0.345
ANA positivity, (*n* = 114) *n* (%)	62 (96.9)	47 (94.0)	0.657
Currently on IS (yes), *n* (%)	22 (33.8)	14 (27.5)	0.469
Currently on Prostanoids (*n* = 115) (yes), *n* (%)	24 (37.5)	6 (11.8)	0.002

Data are expressed as *n* (%) and mean ± SD (or median, IQR). See Table 1 for abbreviations.

**Table 4:** AHA positivity and follow-up events.

	AHA Positive *n* = 56	AHA Negative *n* = 59	*p*-Value
Any cardiac event, *n* (%)	7 (11.9%)	8 (14.6%)	0.785
Pulmonary arterial hypertension, *n* (%)	0 (0)	1 (1.7)	>0.999
Right heart failure, *n* (%)	1 (1.8)	0 (0)	0.482
Left heart failure, *n* (%)	2 (3.6)	0 (0)	0.234
Non sustained ventricular tachycardia, *n* (%)	2 (3.6)	1 (1.7)	0.616
Implantable cardioverter defibrillator for sustained ventricular tachycardia, *n* (%)	1 (1.8)	2 (3.4)	>0.999
Coronary artery disease, *n* (%)	1 (1.8)	2 (3.4)	>0.999
Any other arrhythmia (yes), *n* (%)	6 (10.7)	2 (3.4)	0.123
Death, *n* (%)	5 (8.9)	0 (0)	0.021

Data are expressed as *n* (%).

## Data Availability

Data will be available upon reasonable request to the corresponding author.

## Supplementary Materials

The following are available online at https://www.mdpi.com/article/10.3390/diagnostics11112165/s1. Table S1: AIDA positivity and follow-up events.

## Abbreviations

ACA	anti-centromere antibodies
AHA	anti-heart antibody
AIDA	anti-intercalated disk antibodies
ANA	anti-nuclear antibodies
ATA	anti-topoisomerase I antibodies
CAD	coronary artery disease
CI	confidence interval
CMR	cardiac magnetic resonance
DLco	diffusion capacity of the lung for carbone oxyde
DUs	digital ulcers
ECG	electrocardiogram
Echo	echocardiography
FVC	forced vital capacity
HF	heart failure
HInv	heart involvement
HR	hazard ratio
HRCT	high-resolution computed toography
ICD	implantable cardiac defibrillator
ID	immunofluorescence
IHD	ischemich heart disease
ILD	interstitial lung disease
IS	immunosuppression
LGE	late gadolinium enhancement
LVEF	left ventricle ejection fraction
mRSS	modified Rodnan skin score
NBD	normal blood donors
NICD	non-inflammatory cardiac disease
nSVT	non-sustained ventricular tachycardia
NT-proBNP	type B pro-natriuretic peptide
NVC	nailfold videocapillaroscopy
NYHA	New York heart association
OR	odds ration
PAPs	pulmonary artery pressure in systole
PH	pulmonary hypertension
RNA	ribonucleic acid
RVEF	right ventricle ejection fraction
SD	standard deviation
SIDs	systemic immune-mediated diseases
SSc	systemic sclerosis
STIR	short T inversion recovery
TAPSE	tricuspid Annulus Plane Systolic Excursion
VEDOSS	very early diagnosis of systemic sclerosis
