# Supplementary material for: Serum Organ-Specific Anti-Heart and Anti-Intercalated Disk Autoantibodies as New Autoimmune Markers of Cardiac Involvement in Systemic Sclerosis: Frequency, Clinical and Prognostic Correlates
Source: Diagnostics (Basel). 2021 Nov 22;11(11):2165. doi: 10.3390/diagnostics11112165 (PMC8625508; doi:10.3390/diagnostics11112165)
Supplement: Supplementary file 1 [file diagnostics-11-02165-s001.zip › diagnostics-1445762-supplementary/diagnostics-1445762-supplementary.pdf]

Supplemental Material

Supplementary Table SI. AIDA positivity and follow-up events.

	AIDA positive n=64	AIDA negative n=51	p-value
Clinical worsening of cardiac symptoms, n (%)	8 (12.5)	7 (13.7)	>0.99
Pulmonary arterial hypertension, n (%)	0 (0.0)	1 (2.0)	0.44
Right heart failure, n (%)	0 (0.0)	1 (2.0)	0.44
Left heart failure, n (%)	1 (1.6)	1 (2.0)	>0.99
Non sustained ventricular tachycardia, n (%)	2 (3.1)	1 (2.0)	>0.99
Implantable cardioverter defibrillator, n (%)	1 (1.6)	2 (3.9)	0.58
Coronary artery disease, n (%)	1 (1.6)	2 (3.9)	0.58
Any arrhythmia (yes), n (%)	6 (9.4)	2 (3.9)	0.22
Death, n (%)	4 (6.2)	1 (2.0)	0.38

Data are expressed as n (%) and mean±SD (or median, IQR). See Table 1 for abbreviations
